# Philadelphia Hospital

**Published:** 1844-01-27

**Authors:** 


### CLINICAL LECTURES AND REPORTS.

### PHILADELPHIA HOSPITAL.

### CLINIC OF THE JEFFERSON MEDICAL COLLEGE.*

*January* 6, 1844.

BY PROFESSOR DUNGLISON.

### APOPLEXY AND PARALYSIS.

The Professor commenced with some remarks relative to the pathology of apoplexy, which had formed the subject of his preceding lecture. He believed it to arise in most cases from pressure—the consequence of an effusion of blood or of serum or of hyperæmia in some part of the great nervous centres; and, owing to the decussation of the nervous fibres, the paralytic phenomena, in encephalic paralysis, being observed upon the side opposite to the seat of the primary affection. That the nature of the effusion, whether of blood or serum, might determine in a measure the greater or less permanency of the resulting paralysis; in the latter case, absorption being much more likely to ensue rapidly than in the other. That apoplexy and paralysis may obtain, causing the death of the patient, without presenting any apparent lesion on dissection. Such cases may be due to the disappearance of pre-existing hyperæmia or effusion, previous to examination, or they may be owing, he believed, to an inappreciable modification of the neurine itself, which might give rise to phenomena similar to those that are usually induced by pressure. In such case we may have true nervous apoplexy or paralysis. This form of paralysis may wholly disappear. It occurs in nervous hysterical subjects, and appears to bear the same relation to hysteria that catalepsy does, and may properly be classed with it amongst the neuroses.

The lecturer suggested that the state known as "*magnetic*," may be a nervous condition, referable to this class, in which peculiarly susceptible persons are subjected to nervous impressions, conveyed through the medium of the senses, by which they are rendered distinctly sensitive to certain influences, whilst to ordinary mechanical irritation they may be more or less insensible. In all such cases the strange phenomena must be owing to a modification of the neurine; yet, formidable as these would strike the observer, had he not learned from experience that they are of no consequence, the neurine soon recovers itself.

### PARAPLEGIA.

Cases of paralysis frequently result from injury to the spinal cord, constituting paraplegia, in which motion or sensation, either singly or together, may be impaired or destroyed.

* [For this report, we are indebted to Dr. C. J. Clark, of Alabama, and Messrs. E. Squibb, of Philadelphia, and Samuel G. White, of Georgia, members of the hospital class.—ED.]

[Here the Professor introduced a diagram upon the black board, illustrative of the origin of the spinal nerves; demonstrating that the motor filaments arise from the anterior, and the sensitive from the posterior columns of the spinal marrow, by which the practicability of either the motor or the sensitive function being destroyed, without the other being implicated, was clearly exhibited.]

A patient was here introduced who, some years ago, was thrown from a stage, and lost the power of motion of his inferior extremities, but retained that of sensation. When he executes an effort of volition, which he did before the class, the locomotive influence was seen to be distributed in an irregular manner. When he attempted to raise his inferior extremities there was a spasmodic trembling or jerking of the muscles. He lost the use of his limbs immediately upon receiving the injury. Fell on his side. Has never had perfect control over his urinary organs since. Cannot retain much urine at a time. The same state existed with regard to the fæces, but he is now better in that respect. In this case, the action of the reflex system of nerves was implicated, and the improvement has been chiefly in them. The lecturer thought it probable, that there was such an injury of the anterior column that no advantage could at any time have been derived from internal treatment. He has been subjected to various systems of medication, counter-irritation, strychnia, etc., but the lesion cannot be remedied.

Another case, of a man aged 38, was introduced. Three years ago he fell from a scaffold, striking his back. He was confined to bed for four months. Had no control over his fæces or urine during this period. Sensation and locomotion were lost in both legs. Both columns were injured in this case. He was for a long time impotent, but his virile powers are slowly returning. The patient's back was examined before the class, and he was made to walk about, which he did with a peculiar tottering gait, crossing one foot over the other. Sensation still remains imperfect below the seat of the injury. A prominence was distinctly seen in one portion of the lumbar region of the spine, and a depression immediately beneath it. The depression was at the place where he was struck. When his back was examined, by pressing with the fingers from above to below, sensation existed until they reached the point of depression. The paraplegic phenomena are not as fully marked as we often meet with them. A common case of paraplegia was described. The lecturer remarked, that when the symptoms he detailed begin to show themselves, we must immediately direct our attention to the spinal marrow; for, after paraplegia is once completely developed, the treatment is often of but little avail. When it comes on from an accident it is generally developed at once, and there is less chance of cure, unless surgery can suggest a remedy. We have, in such cases, to trust principally to the recuperative powers; but when it arises spontaneously we may employ counter irritants, as moxas, blisters, strychnia or brucia, internally or endermically, and at times with benefit.

### CHOREA.

The lecturer then passed to the consideration of a case clearly belonging to the neuroses, and introduced a young female affected with chorea, a strongly marked case, in which there were most striking irregular and involuntary muscular contractions, causing a jerking about of the limbs and body, and a frightful distortion of countenance. "Here," said he, "there is hardly a single voluntary muscle that is not thrown into violent irregular contraction." He proceeded to detail her case. She was 21 years of age, and had her catamenia last week for the first time, without any improvement. Her father was subject to epileptic fits. Both epilepsy and chorea are marked by great impressibility of the nervous system. Chorea he thought to be not a very common disease, certainly not in this city. Dr. Reese, of New York, says he treated two hundred cases with arsenic alone, leaving us to infer that others had been treated by other agents. Yet the lecturer had not seen more, perhaps, than thirty well marked cases in all. This is the only case in the wards at this time. There were four or five during the last year, and those were very slight. Chorea is generally first manifested by what is supposed to be a vicious habit on the part of the child, as lolling out the tongue, and throwing the head to one side, and dragging one leg in a strange manner; but it is soon recognized. It would seem, that there must be some lesion of the nervous centres in these cases, yet it almost always passes away. The disease occurs more frequently in young children, and sometimes runs into epilepsy. When it commences in childhood, it usually subsides at puberty. Great torpor of the bowels and constipation almost always exist in these neurotic affections, and require the use of brisk purgatives, which are likewise valuable revellents. These, combined with tonics—as the preparations of iron or zinc, and the cold shower bath, usually effect a cure. The nervous system is in a state of great mobility, and we have to give new tone to it. The properties and mode of operation of cimicifuga were described. It is an acro-narcotic in large doses; and there are cases of chorea in which its use has been attended with advantage. Arsenic has been greatly recommended by some—the Liquor Arsenicalis, for example, in the dose of four or five drops, twice a day, to children six or eight years of age, and continued for a few weeks; its effects being carefully watched, and when certain symptoms, as puffiness of the face, &c., appear, it must be discontinued. Arsenic has had as good an effect in the lecturer's hands as tonics generally. Nitrate of silver is greatly extolled by many practitioners. The disease has been treated by a few with bloodletting; but with the lecturer's views of the pathology of the disease this treatment does not appear philosophical. It is one of the neuroses, characterized by great impressibility of the nervous system. Where it lasts beyond puberty, it is very unmanageable, and more apt to terminate in epilepsy. There is great danger that it may end in dementia. It is sometimes feigned in hospital, and even in private practice, and is by no means easy of detection in all cases. The test of absence of motion of the affected muscles during sleep cannot be relied upon, as it frequently exists in true cases of chorea.

The lecturer concluded with a remarkable example of feigned disease, which fell under his notice many years ago, in a young lady who was unhappy at school, and assumed the symptoms of chorea of the left arm. She was subjected to every form of treatment, and to agencies of a severe kind, yet the affection did not yield, and its true nature was only detected by directing her nurse to watch through the keyhole of the door of her chamber, whilst she was alone within, and thought herself unobserved. At such time there was no motion; but when the nurse made a slight noise at the door, it was seen to be renewed.

